# Research on an Artificial Lateral Line System Based on a Bionic Hair Sensor with Resonant Readout

**DOI:** 10.3390/mi10110736

**Published:** 2019-10-29

**Authors:** Bo Yang, Ting Zhang, Zhuoyue Liang, Chengfu Lu

**Affiliations:** 1 School of Instrument Science and Engineering, Southeast University, Nanjing 210096, China; 220183214@seu.edu.cn (T.Z.); 220183262@seu.edu.cn (Z.L.); 220162752@seu.edu.cn (C.L.); 2 Key Laboratory of Micro-Inertial Instrument and Advanced Navigation Technology, Ministry of Education, Nanjing 210096, China

**Keywords:** bionic hair sensor with resonant readout, artificial lateral line system, oscillatory air flow velocity

## Abstract

Inspired by the lateral line system of fish, an artificial lateral line system based on bionic hair sensor with resonant readout is presented in this paper. An artificial lateral line system, which possesses great application potential in the field of gas flow visualization, includes two different sensors: a superficial neuromast and a canal neuromast flow velocity sensor, which are used to measure the constant and oscillatory air flow velocity, respectively. The sensitive mechanism of two artificial lateral line sensors is analyzed, and a finite element simulation is implemented to verify the structural design. Then the control circuit of the artificial lateral line system is designed, employing a demodulation algorithm of oscillatory signal based on the least mean square error algorithm, which is used to calculate the oscillatory air flow velocity. Finally, the experiments are implemented to assess the performance of the two artificial lateral line systems. The experimental results show that the artificial lateral line system, which can be used to measure the constant and oscillatory air flow velocity, has a minimum threshold of 0.785 mm/s in the measurement of oscillatory air flow velocity. Moreover, the artificial canal neuromast lateral line system can filter out low-frequency disturbance and has good sensitivity for high-frequency flow velocity.

## 1. Introduction

The lateral line system is a primitive sensory organ in fish. Inspired by this system, researchers have reported multiple artificial lateral line systems based on MEMS technology for the velocity measurement [1,2]. The artificial lateral line system usually consists of two kinds of sensors, one a superficial neuromast flow velocity sensor, and the other a canal neuromast flow velocity sensor. An artificial lateral line system, which can measure the constant and oscillatory flow in different media, has great practical value in insect robots, unmanned underwater vehicles or other advanced technological products. Krijnen et al. proposed superficial hair flow velocity and acceleration sensors based on capacitive readout, and the sensor has good directional characteristics [3,4]. A superficial microhydraulic amplification hair flow velocity sensor with capacitive sensing was also studied in [5]. The sensor can measure the air flow velocity of 0–15 m/s. Chen et al. developed a piezoresistive hair sensor for the underwater artificial lateral line system, which can measure the oscillatory flow velocity in water generated by the vibration of dipole oscillator [6,7]. The amplitude of oscillatory flow velocity can be analyzed by FFT (fast Fourier transform) algorithm of output frequency signal. The experimental method of FFT based on a spectrum analyzer that is used to observe the corresponding amplitude of the oscillatory flow velocity signal is inefficient and so it is hard to miniaturize the overall system. Kottapalli et al. verified the high-pass filtering characteristics of artificial lateral line systems that can filter constant flow velocity signal and be fabricated by piezoelectric hair flow velocity sensor [8,9]. A photoelectric hair flow sensor was proposed by Bleckmann et al. The influence of canal, hole and hair structure parameters on the performance of the artificial lateral line system is analyzed, which provides a reference for subsequent design of the artificial canal neuromast lateral line system [10,11,12].

This paper focuses on the design of an artificial lateral line system based on the bionic hair sensor with resonant readout. The bionic hair sensor with resonant readout which has high sensitivity in flow measurement was proposed by Yang et al. [13]. The main purpose of this paper is to investigate the array characteristics of hair sensors for the artificial lateral line system. The artificial lateral line system, which consists of two types of sensors, the superficial flow sensor and the canal flow sensor, can accurately measure the constant and oscillatory air flow velocity. The canal artificial lateral line system shows good high-pass filtering characteristics. The weak oscillatory air flow can also be measured, even if it is submerged in a strong steady state flow. In Section 2, the structure principle and the simulation of bionic hair sensor with resonant readout are described briefly. In Section 3, the measurement circuit and the demodulation algorithm are presented. Then, the experimental results are illustrated in Section 4. Concluding remarks are finally given in the last section.

## 2. Structure Design and Simulation

### 2.1. Structure Design

**Figure 1:**
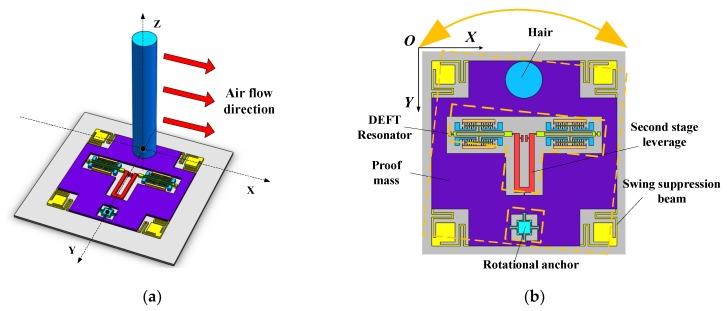
(**a**,**b**) Structure of the bionic hair sensor with resonant readout.

The structure of the bionic hair sensor with resonant readout is shown in Figure 1 [13]. The sensor consists of three parts: upper hair, middle silicon microstructures and lower glass substrate, as shown in Figure 1a. The silicon microstructure is bonded to the glass substrate through MEMS processing. Employing UV adhesives and micro-assembly, the hair is solidified to the middle silicon microstructures. The hair of acrylonitrile butadiene styrene (ABS) polymer with a density of 1.05 g/cm^3^ has light weight and good comprehensive performance, which is beneficial for reducing the influence of hair mass on the flow rate sensitivity. As shown in Figure 1b, when the hair is dragged by the external air flow in the X-axis direction, the microstructure frame is driven by the hair to deflect an angle around the rotational anchor. The drag force transmitted through the microstructure is exerted on the axial direction of two tuning fork resonators. The natural frequency of the two resonators changes when they are subjected to the axial external force [14]. The frequency of one resonator increases, while the frequency of the other decreases. By measuring the frequency difference between the two resonators, the drag force of hair and the external flow velocity can be deduced. The structure parameters are shown in Table 1.

According to the principle of resonant detection, the frequency difference variation of the two resonators is [13]:

(1) $$\Delta f=\frac{1}{2}tf_{0}F_{out}=\frac{1}{2}tf_{0}A\eta C_{D}\rho RL_{H}u^{2}$$

where *t* is a small constant related to the structure of the resonant beam and *t = λL*^2^*/ehw*^3^. *λ* is a coefficient related to the resonator, *L* is the length of the resonator, *h* is the thickness of the resonator, *w* is the width of the resonator beam, and *e* is the Young’s modulus. *f*_0_ is the natural resonant frequency of the resonator without the external force, *A* is the magnification of the two-stage leverage mechanism, *η* is the attenuation coefficient, *C_D_* represents the drag coefficient, which is related to the geometry and surface roughness of hair, *ρ* denotes the density of air, *R* is the diameter of the hair, *L_H_* is the length of the hair, and *u* is the flow velocity in air.

Furthermore, the hair sensor can measure the oscillatory flow velocity. The transferred drag force and the frequency difference variation of the resonator are also oscillatory quantities [15]. In order to simplify the calculation, the drag force of hair *F_s_* in the excitation of oscillatory air flow can be expressed as follows [16]:

(2) $$F_{S}(t)=U\cdot |Z_{S}|\sin(\omega t+\eta_{S})$$

where *Z_s_* represents the mechanical impedance of hair, relating to the hair structure parameters, the input frequency of oscillatory air flow, properties of the fluid medium, etc. *η_s_* represents the phase difference between the input signal and the drag force. In the oscillatory flow field, the drag force of the hair is the oscillatory force and the amplitude of the oscillatory force is proportional to the amplitude of the oscillatory flow velocity. In order to implement the experiment of the oscillatory flow velocity signal for the artificial lateral line system, the oscillatory flow velocity is generated by the speaker with AC excitation. Simultaneously, sound pressure is calibrated by the microphone. When the sound wave propagates, the medium particle will vibrate back and forth near its equilibrium position. This kind of vibration speed is called particle speed, which is related to the strength of the sound wave. The relationship between the medium particle vibration velocity of plane wave *V_x_* and the effective sound pressure *P_e_* is [17]:

(3) $$V_{x}=\frac{P_{e}}{\rho_{0}c_{0}}\sin(\omega_{1}t-\frac{\omega_{1}}{c_{0}}x)$$

where *ρ*_0_ is the air density of 1.29 kg/m^3^, *c*_0_ is the acoustic velocity of 340 m/s in the air, *ω*_1_ is the vibration frequency, and *x* is the distance from particle to acoustic source. The velocity amplitude of particle is proportional to the effective sound pressure.

**Figure 2:**
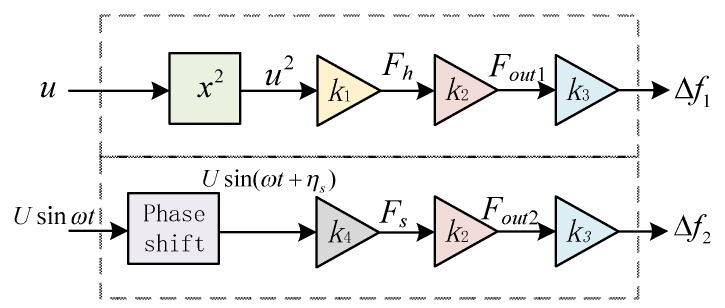
Relationship between air flow velocity and output signal of sensor.

The relationship between the air flow velocity and the output signal of the hair sensor is shown in Figure 2. The elastic force *F_out_* transmitted by the hair through the silicon microstructure is applied to the left and right resonator, respectively. Theoretical analysis shows that the elastic force *F_out_* is proportional to the drag force of hair, while the natural frequency variance of the resonator is approximately proportional to the axial force *F_out_*. In Figure 2, *k*_1_ = *C_D_ρRL_H_*, *k*_2_ *= Aη*, *k*_3_ *=* (1/2)*tf*_0_, *k*_4_ *= |Z_s_|. ∆f*_1_ represents the output frequency difference of two resonators in the constant air flow velocity of *u*, while *∆f*_2_ represents the output signal of sensor in the oscillatory air flow velocity of *Usinωt*.

### 2.2. Artificial Lateral Line System

**Figure 3:**
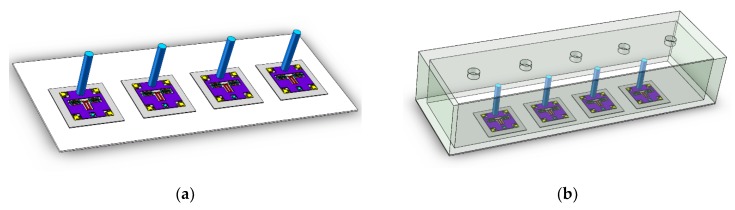
Schematic diagram of artificial lateral line system. (**a**) Superficial neuromast flow velocity sensor; (**b**) Canal neuromast flow velocity sensor

The schematic diagram of two artificial lateral line systems is shown in Figure 3.

The first artificial lateral line system, which is a superficial neuromast flow velocity sensor, consists of an array of multiple bionic hair sensors with resonant readout, as shown in Figure 3a. The second artificial lateral line system, which is a canal neuromast flow velocity sensor, is composed of an array of multiple bionic hair sensors with resonant readout and a peripheral canal cover, as shown in Figure 3b. The sensitive axes of four bionic hair sensors with resonant readout are in a straight line and are located in the canal. According to the traditional hydrodynamics, the drag force on the hair mainly comes from the differential pressure resistance. The differential pressure resistance is due to the different flow velocity between the front area and rear area of the hair, in which low velocity results in the lowpressure area at the back of the hair, and high velocity results in the high-pressure area at the front of the hair [13]. Ulteriorly, a significant pressure difference between the front and the rear area of the hair can emerge. There is a row of holes above the canal to connect the internal media with the external air. When the artificial lateral line system of the canal neuromast flow velocity sensor is subjected to the oscillatory air flow field, the pressure difference between the two adjacent holes brings about the oscillatory vibration of the air inside the canal, which drives the oscillatory oscillation of the bionic hair sensors [18].

### 2.3. Simulation

**Figure 4:**
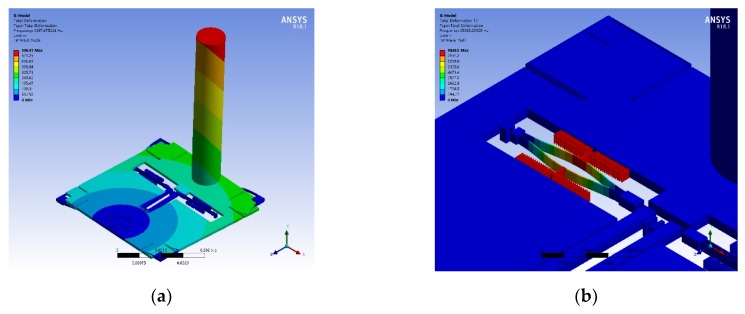
Simulation modes of bionic hair sensor with resonant readout. (**a**) Sensitive mode; (**b**) Anti-phase resonant mode of the resonator.

The finite element modal simulation of the bionic hair sensor is implemented with a hair length of 6 mm and a diameter of 1 mm. Figure 4a is the first-order modal diagram of the bionic hair sensor with a modal frequency of 1287.5 Hz, which is the sensitive mode of the bionic hair sensor for the air flow velocity and mainly the rotational motion of the hair around the anchor. The decrease of the sensitive mode frequency can significantly increase the sensitivity. However, sensitive mode frequency is also comprehensively compatible with the bandwidth requirements. Figure 4b shows the anti-phase resonant mode of the resonator, which is the operating mode of the resonator with a resonant frequency of 25686.3 Hz. The natural resonant frequency of the resonator will shift under the excitation of external axial stress.

**Figure 5:**
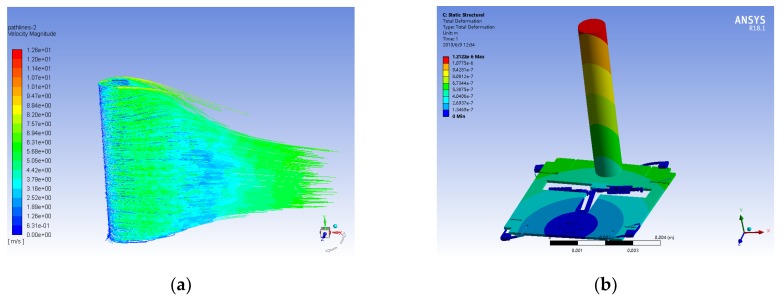
Flow velocity simulation of 10 m/s in a superficial neuromast flow velocity sensor. (**a**) Fluid velocity path-line of the hair surface; (**b**) Displacement simulation of the hair sensor.

The flow velocity simulation of the entire structure is implemented to verify the response characteristics caused by flow rate excitation, shown in Figure 5.

First, the fluid-solid coupling simulation analysis is carried out by ANSYS to obtain the fluid velocity path-line around the hair surface at an air flow rate of 10 m/s, as shown in Figure 5a. Then the pressure of the hair surface is imported to the static analysis module to obtain the displacement of the entire structure, as shown in Figure 5b. The finite element analysis results illustrate that the maximum displacement of the hair is 1.212 µm at the top of the hair, while the maximum displacement of the proof mass is 0.586 µm, with a rotation angle of 1.673 × 10^−6^° around the rotational anchor. The maximum stress of a single resonator in the axial direction is 2.212 MPa in the excitation of air flow rate of 10 m/s, which arouses a natural frequency change of 149 Hz in the resonator. The input flow velocity will drive the rotation of proof mass, causing axial stress to be applied to the internal resonators, and will eventually lead to the natural frequency variation of resonators.

**Figure 6:**
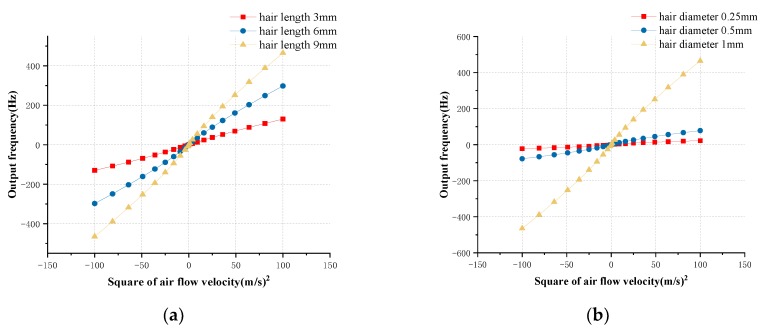
The influence simulation of hair parameters on input and output characteristics. (**a**) The output frequencies versus square of input air flow velocity in different hair lengths of 3 mm, 6 mm, and 9 mm (the hair diameter is 1mm); (**b**) The output frequencies versus square of input air flow velocity in different hair diameter of 0.25 mm, 0.5 mm, and 1 mm (the hair length is 9 mm).

Different hair structures and material parameters result in different mechanical properties. We have the flexibility to choose different hair structures and materials to ensure optimum performance. Concerning hairs of different lengths and diameters, the simulation is carefully implemented to optimize the performance of the bionic hair sensor with resonant readout at different flow velocities. The output frequency variation curve with different hair lengths of 3 mm, 6 mm, and 9 mm is shown in Figure 6a at different input air flow velocities. Since the output frequency of the hair sensor is approximately linear with the square of the input air flow velocity. The relationship between the output frequency and the square of input air flow velocity is linearly fitted. The fitting results indicate that the sensor sensitivities in different hair length of 3 mm, 6 mm and 9 mm are approximately 1.313 Hz/(m/s)^2^, 2.992 Hz/(m/s)^2^, and 4.669 Hz/(m/s)^2^, respectively. Similarly, the influence of different hair diameters on the output response of the sensor is simulated. The output frequency variation curve at different input air flow velocities in different hair diameters of 0.2 mm, 0.5 mm and 1 mm is shown in Figure 6b. The fitting result indicates that the sensor sensitivities in different hair diameters of 0.2 mm, 0.5 mm, and 1 mm are approximately 0.220 Hz/(m/s)^2^, 0.787 Hz/(m/s)^2^, and 4.669 Hz/(m/s)^2^, respectively. The simulation results show that the output frequency variation of the hair sensor is proportional to the square of the air flow velocity. The increase of hair length and diameter can significantly increase the mechanical sensitivity of the sensor, which is basically consistent with theoretical analysis. The sensitivity of the hair sensor is positively correlated with the length and diameter of the hair, but it is not strictly proportional, as there is a certain deviation from the theoretical results. The main reason for this is the simulation error of the boundary layer thickness and the drag coefficient *C_D_.* In practical applications, an ABS polymer hair with a length of 9 mm and diameter of 1 mm is adopted for maximum mechanical sensitivity.

**Figure 7:**
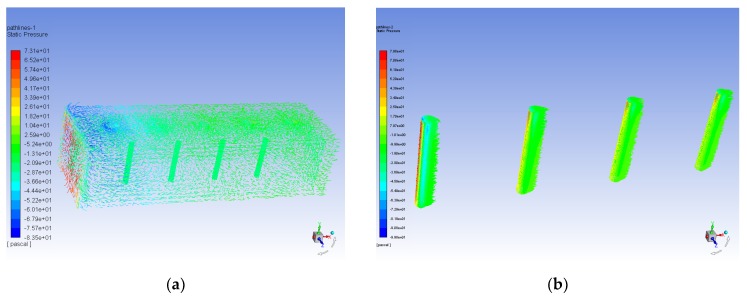
Pressure simulation comparison of hair surface in the artificial lateral line system at flow velocity field of 10 m/s. (**a**) Pressure path-lines diagram of canal neuromast flow velocity sensor; (**b**) Pressure path-lines diagram of superficial neuromast flow velocity sensor.

The preceding simulation mainly verified the flow velocity measurement function of the superficial neuromast flow velocity sensor in the artificial lateral line system. In addition to the superficial neuromast flow velocity sensor, the characteristics of the canal neuromast flow velocity sensor are simulated by the second artificial lateral line system. In order to verify the function of the canal neuromast flow velocity sensor to suppress low-frequency or constant flow velocity, a 10 × 10 × 60 cm flow field is constructed. The simulation of one-way fluid-solid coupling is implemented in the air flow velocity of 10 m/s. The stress acting on the canal neuromast flow velocity sensor is transmitted into the static force module to perform a static force analysis. Figure 7 shows the pressure response of the hair surface in the superficial neuromast and canal neuromast flow velocity sensors.

As shown in Figure 7a, the canal blocks the constant or low-frequency air flow in the canal neuromast flow velocity sensor, so the hair sensor is less affected. However, the superficial neuromast flow velocity sensor is significantly affected by the external constant or low-frequency air flow, shown in Figure 7b. The simulation results illustrate that the stress of the hair surface in the canal neuromast flow velocity sensor is only 2.59 Pa, while that in the superficial neuromast flow velocity sensor is 79.8 Pa. The hair is isolated from the external interference of low-frequency fluid due to the low-frequency filter function of the canal neuromast flow velocity sensor. However, the air in the artificial lateral line canal is still connected to the outside through the hole on the canal. The hair array in the canal neuromast flow velocity sensor can receive the pressure difference generated by the high-frequency oscillatory fluid. The simulation demonstrates that the canal neuromast flow velocity sensor of the artificial lateral line system can suppress constant or low-frequency fluid interference and has a high-pass filtering function. This feature allows the fish to avoid predation by its natural enemies, since short-term explosive movements will produce high-frequency disturbances in the surrounding fluids when enemies begin to attack. The canal hair array on the fish’s body are only sensitive to high-frequency disturbances, while the usual natural fluids in the surroundings have a low frequency and can be filtered out by the canal hair array. As the finite element simulation software can’t simulate the characteristics of oscillatory fluid, the experiment and verification of the oscillatory flow in the canal neuromast flow velocity sensor are completed in the experimental part in Section 4.

## 3. Measurement Circuit and Demodulation Algorithm

### 3.1. Measurement Circuit

**Figure 8:**
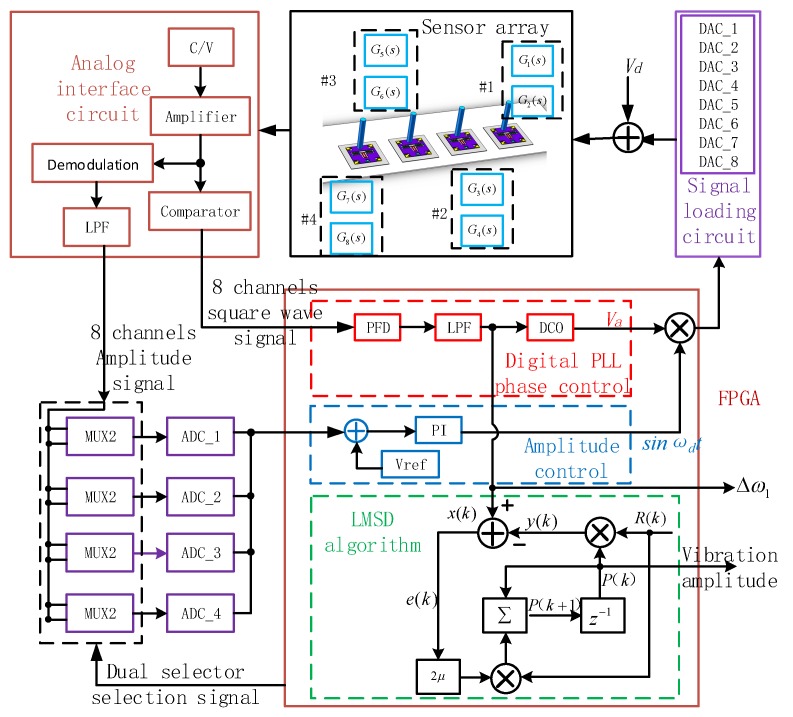
Measurement and control circuit block diagram of the system.

In order to realize the signal measurement of the artificial lateral line system, a closed-loop control and measurement system for the bionic hair sensor with resonant readout array is designed, as shown in Figure 8. Each individual bionic hair sensor contains two resonators that are electrostatically driven and capacitively detected [19]. When the resonator is operating in the resonant mode, the resonant beam has the largest vibration displacement and the phase of the output displacement lags behind that of the input drive voltage signal by 90°. The differential capacitance is developed between the detection comb on the resonant beam and the fixed comb. The vibration state of the resonant beam, including the vibration frequency, the vibration displacement and the phase difference between the input and output, can be obtained by detecting the variation of the differential capacitance. The peripheral analog circuit converts the capacitance signal into a voltage signal, and performs amplification and demodulation processing to obtain eight amplitude signals through the low-pass filters. Each two amplitude signals are input into a two-way selector that adopts time division multiplexing to share one Analog to Digital Converter (ADC) chip. The eight-channel amplitude signal is quantized and transmitted to a field programmable gate array (FPGA) for subsequent processing through four ADC chips. Simultaneously, the amplified signals of differential capacitance are converted into eight-channel square waves through the comparators and transmitted to the FPGA, which mainly acquire the phase information of the differential capacitance signal. The digital phase-locked loop (PLL), which includes a phase frequency detector (PFD), a LPF, and a digitally controlled oscillator (DCO), is designed to track the frequency and phase of the input signal in the PFGA. The phase-locked loop has a phase shift of exactly 90° in the locked condition, which can be used to implement a closed-loop self-excitation drive. An amplitude control loop that includes a proportional integral (PI) controller and a multiplier is used to maintain the stable vibration amplitude of the resonator. In addition to the digital phase-locked loop and the amplitude control loop, the least mean square demodulation (LMSD) algorithm is implemented in the FPGA, which extracts the oscillatory amplitude of the frequency control word in the loop filter output. This amplitude represents the vibratory amplitude of hair in the excitation of external oscillatory air flow.

**Figure 9:**
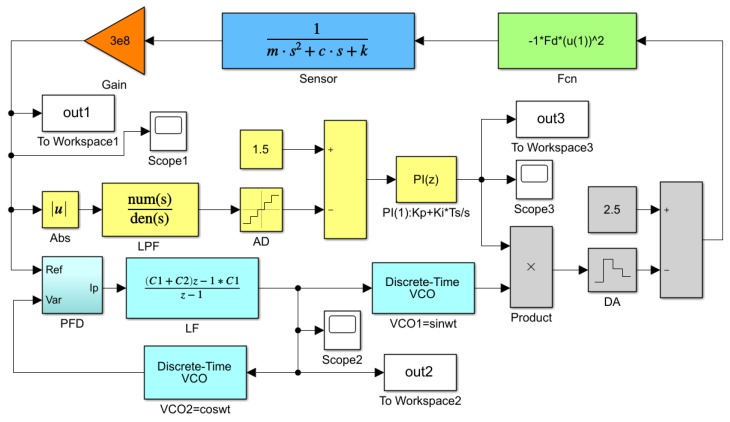
Simulink simulation model of the measurement and control circuit.

**Figure 10:**
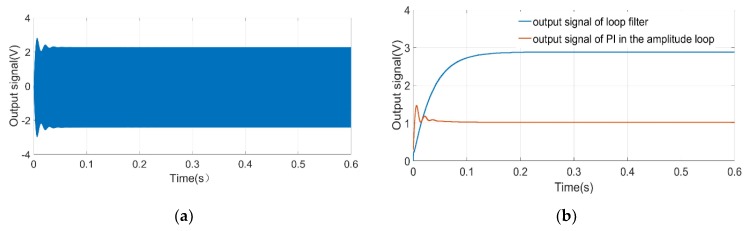
Waveform diagram of key signals (**a**) output signal of pre-amplifier (**b**) output signal of loop filter and output signal of PI in the amplitude loop

In order to verify the feasibility of the control circuit scheme, a simulation model (shown in Figure 9) is built by Simulink. The module of *‘Fcn’* set in the system converts the voltage signal into an electrostatic force signal, and then the signal is applied to the input terminal of the resonator to realize the torque function. Based on the sensor parameters and system simulation parameters in Table 2, the system simulation is implemented to obtain the waveform diagram of the key signals. Figure 10 illustrates the simulation output signal curve of the pre-amplifier, loop filter, and PI in the amplitude loop. Three curves gradually approach stability after a short period of fluctuation. The system can achieve the closed-loop control of phase and amplitude within 0.2 s. The Simulink simulation results shown in Figure 10 demonstrate the effectiveness of the control system.

### 3.2. Demodulation Algorithm

The constant flow velocity can be directly obtained by measuring the frequency of the control system output. However, a special signal demodulation algorithm is needed to acquire the amplitude of the oscillatory signal if the input is oscillatory air flow. The LMSD algorithm is employed to achieve amplitude extraction of oscillatory signals in the excitation of oscillatory flow velocity.

Using the coordinate rotation digital computer (CORDIC) algorithm in the phase-locked loop [20], the orthogonal digital reference signals are simultaneously output:

(4) $$R(k)=\left[\begin{array}{l} s(k)\\ c(k) \end{array}\right]$$

where *s*(*k*) *= sinω_c_t*, *c*(*k*) *= cosω_c_t* and *ω_c_* is the frequency of the oscillatory air flow velocity.

**Figure 11:**
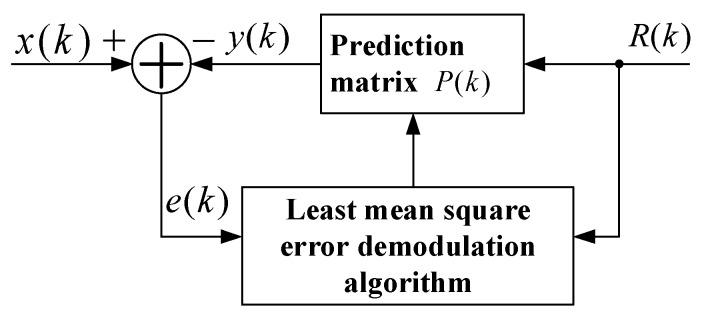
The functional block diagram of the least mean square demodulation (LMSD).

In the oscillatory flow field, the frequency difference signal of two resonators after an infinite impulse response (IIR) high-pass filter can be expressed as 2*A*sin(*ω*_c_*t* + *θ*), and the signal can be expanded to:

(5) $$2A\sin(\omega_{c}t+\theta )=(2A\cos\theta )\sin\omega_{c}t+(2A\sin\theta )\cos\omega_{c}t$$

where *sinω_c_t* and *cosω_c_t* can be directly generated by the internal CORDIC algorithm of the FPGA. In the actual application, the frequency difference signal may be mixed with noise signal. Considering the influence of the noise signal, Equation (6) can be rewritten as:

(6) x(k)=u(k)+n(k)=Q⋅s(k)+I⋅c(k)+n(k)

where *x*(*k*) is the output signal of the sensor through a high-pass filter, *u*(*k*) is the real signal of sensor and *n*(*k*) is the noise signal. *P =* [*Q I*]*^T^* is the prediction matrix. The principle LMSD algorithm is the estimated signal of *x(k)* can be obtained by iterative approximation of the prediction matrix *P* using the reference signal *R(k)*. The principle block diagram is shown in Figure 11.

The estimated error signal *e*(*k*) can be obtained by subtracting the estimated signal *y*(*k*) from the input signal *x*(*k*). The core of the LMSD algorithm is to minimize the square of the estimated error signal *e*(*k*) so that the estimated signal *y*(*k*) is matched with the input signal *x*(*k*) to obtain an optimal prediction matrix *P*, that is *Q =* 2*A*cos*θ*, *I* = 2*A*sin*θ*. The amplitude of the input signal can be expressed as:

(7) $$A=\sqrt{\frac{Q^{2}+I^{2}}{4}}$$

The LMSD algorithm generally uses an iterative method to obtain an optimal prediction vector *P*. The steepest descent method is a commonly used iterative algorithm. The steepest descent method demonstrates that the next predictive vector *P*(*k +* 1) is equal to the previous predictive vector *P*(*k*) plus the proportionally corrected negative gradient:

(8) P(k+1)=P(k)−μ∇(k)

where *μ* is the convergence factor of the steepest descent method and*▽*(*k*) is the estimated gradient.

If the mean square value is substituted by the instantaneous square value of each iteration of the error signal, the estimated gradient can be expressed as follows:

(9) $$\nabla (k)={\left[\frac{\partial e^{2}(k)}{\partial p_{1}(k)}\frac{\partial e^{2}(k)}{\partial p_{2}(k)}\right]}^{T}=\frac{\partial e^{2}(k)}{\partial P(k)}=\frac{\partial {\left[x(k)-P^{T}(k)R(k)\right]}^{2}}{\partial p_{1}(k)}\;=2e(k)\frac{\partial e(k)}{\partial P(k)}=-2e(k)R(k)$$

Then Equation (8) can be expressed as follows:

(10) P(k+1)=P(k)+2μe(k)R(k)

**Figure 12:**
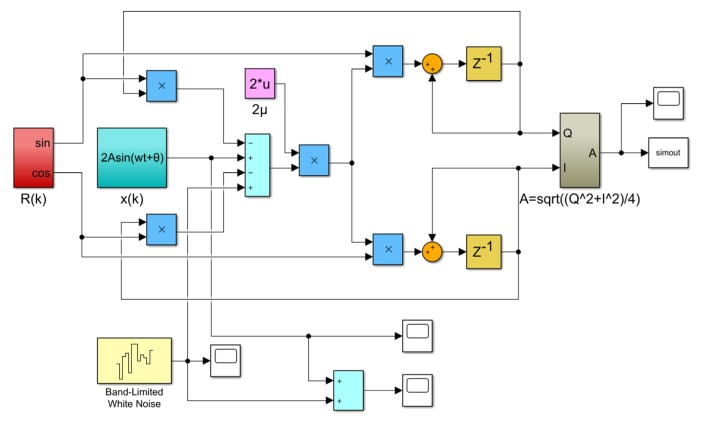
Simulink model of LMSD demodulation algorithm.

Initializing *P*(0) *=* [0 1]*^T^*, and the values of *Q* and *I* can be obtained by iteration of the above formula. Then the amplitude signal of sensor output A can be calculated from Equation (7) based on the values of *Q* and *I.* In order to verify the feasibility and the influence of parameters on performance of the LMSD algorithm, the simulation was implemented by the Simulink platform. Figure 12 is a block diagram of the simulation principle model.

The simulated input signal *x*(*t*) can be expressed as follows:

(11) $$x\left(t\right)=2A\sin(\omega_{c}t+\theta )+n\left(t\right)=2\sin(2\pi \cdot 60t+0.5\pi )+n\left(t\right)$$

where A represents the amplitude of oscillatory frequency difference of the resonator in the excitation of the oscillatory flow velocity and is set to 1, *ω_c_* is the frequency of the oscillatory flow velocity and is set to 2π × 60, *θ* is the phase and is set to 0.5π.

**Figure 13:**
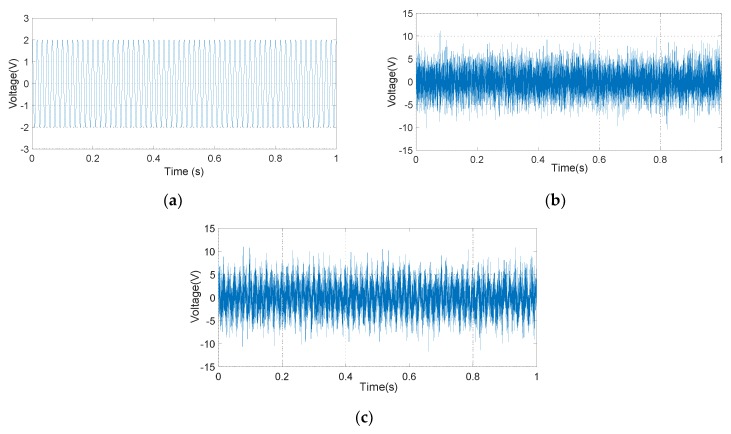
Input signal waveform. (**a**) 60Hz oscillatory signal; (**b**) White Gaussian noise; (**c**) Superimposed input signal.

**Figure 14:**
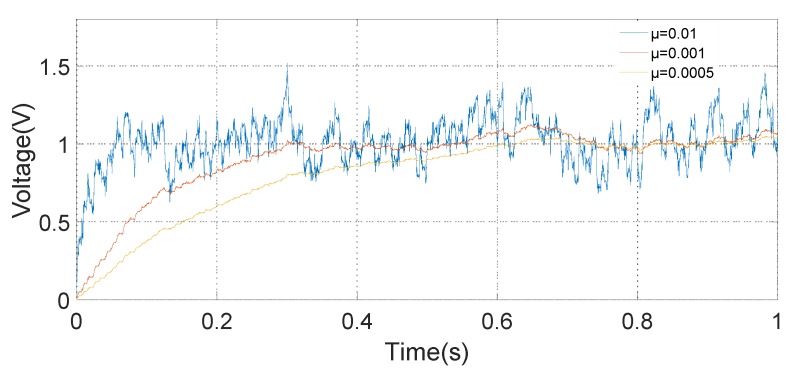
Curve of the demodulation signal amplitude.

The input signal of the simulation system, which is mainly composed of a 60 Hz oscillatory signal and a Gaussian white noise signal, is shown in Figure 13. Because of the excessive Gaussian white noise, it is hard to distinguish the oscillatory signal in the Figure 13c. The amplitude of the demodulation signal based on the LMSD algorithm with *μ* = 0.01, *μ* = 0.001, and *μ* = 0.0005 is shown in Figure 14. The convergence accuracy and speed of the LMSD algorithm are related to the convergence factor *μ*. The simulation results demonstrate that an increase in the convergence factor *μ* will speed up the amplitude convergence rate, but significantly enlarge the amplitude error. Apparently, the decrease of the convergence factor *μ* will be beneficial to reduce the amplitude error. However, the transition time of the amplitude is significantly increased. In a real application, it is necessary to select an appropriate convergence factor *μ* according to the experimental situation. Simultaneously, the simulation results indicate that the LMSD algorithm has strong noise suppression capability: even if the amplitude of the noise signal is up to five times that of the oscillatory signal, the signal amplitude of the oscillatory signal can still be demodulated. The LMSD algorithm has an excellent demodulation performance.

## 4. Experiments

**Figure 15:**
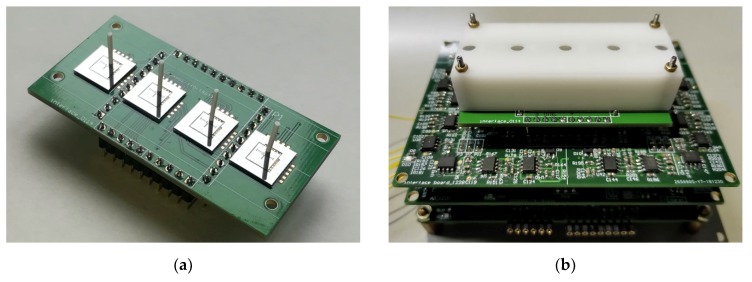
The artificial lateral line system. (**a**) Superficial flow velocity sensor; (**b**) Canal flow velocity sensor

According to the block diagram of the control system in Figure 8, a hardware control circuit of the artificial lateral line system is designed and debugged with the bionic hair sensor. The assembly prototypes of the superficial and canal artificial lateral line system are shown in Figure 15. To verify the correctness of the control system, the performance of two artificial lateral line systems is tested and three experiments are implemented. The artificial lateral line system in the first experiment is excited by the constant air flow velocity. In the second experiment, the artificial lateral line system is excited by the oscillatory air flow velocity. The constant and oscillatory air flow velocity are simultaneously superimposed to excite the artificial lateral line system in the third experiment.

### 4.1. Excitation of Constant Flow Velocity for the Artificial Lateral Line System

**Figure 16:**
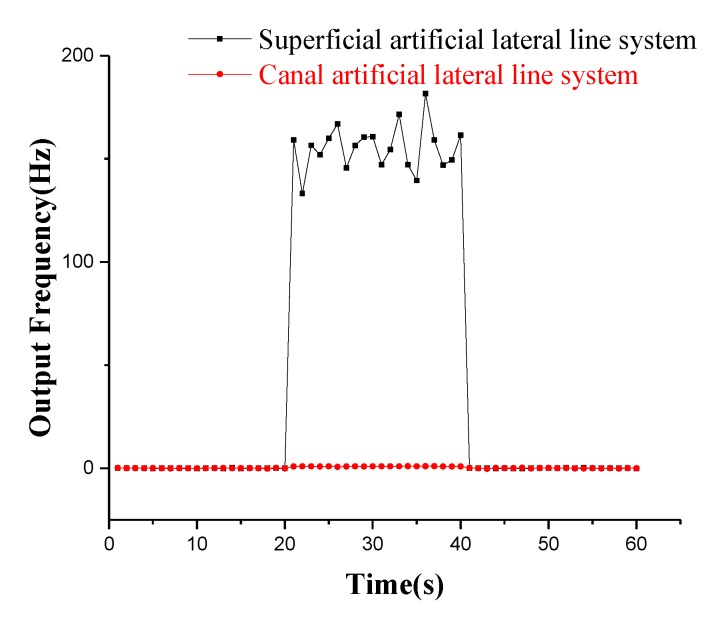
Comparison curve of output signals of two sensors at 5 m/s air flow velocity.

A comparison experiment in the constant air flow velocity is performed on the superficial and canal artificial lateral line system for a 60 s test. First, the two lateral line systems are tested for 20 s without the air flow velocity, then the air flow velocity of 5 m/s is input and preserved for 20 s. Finally, the air flow velocity is cancelled and sustained for the next 20 s. The comparison curve of the two sensors’ output signals at 5 m/s air flow velocity is shown in Figure 16. The two sensors have almost no signal output in the static state. The superficial artificial lateral line system has an approximate frequency output of 155 Hz at the air flow velocity of 5 m/s, and the frequency fluctuation error is mainly caused by the fluctuation of air flow. However, the canal artificial lateral line system completely suppresses the influence of the constant air flow velocity, and the output frequency signal is almost zero. The experimental results demonstrate that the canal artificial lateral line system has a good suppression effect on the constant or low-frequency disturbance air flow velocity.

**Figure 17:**
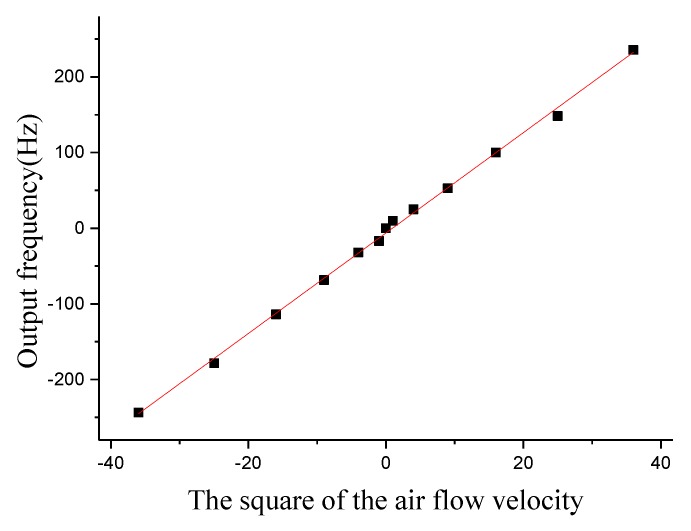
The output signal fitting curve of the superficial artificial lateral line system.

The signal of the superficial artificial lateral line system is measured in the excitation of constant air flow velocity of 1 m/s to 6 m/s. The relationship between the square of the air flow velocity and the output frequency signal is linearly fitted. The output signal fitting curve of the superficial artificial lateral line system is shown in Figure 17. The experimental results indicate that the output frequency signal is basically proportional to the square of the air flow velocity (negative values represent the direction of the input flow rate is the negative direction of X-axis). The scale factors of the four superficial flow velocity sensors are 6.97 Hz/(m/s)^2^, 6.84 Hz/(m/s)^2^, 6.72 Hz/(m/s)^2^, and 6.64 Hz/(m/s)^2^, respectively. It can be seen from the experiment that the superficial artificial lateral line system can detect the constant or low frequency air flow velocity.

### 4.2. Excitation of the Oscillatory Flow Velocity for an Artificial Lateral Line System

**Figure 18:**
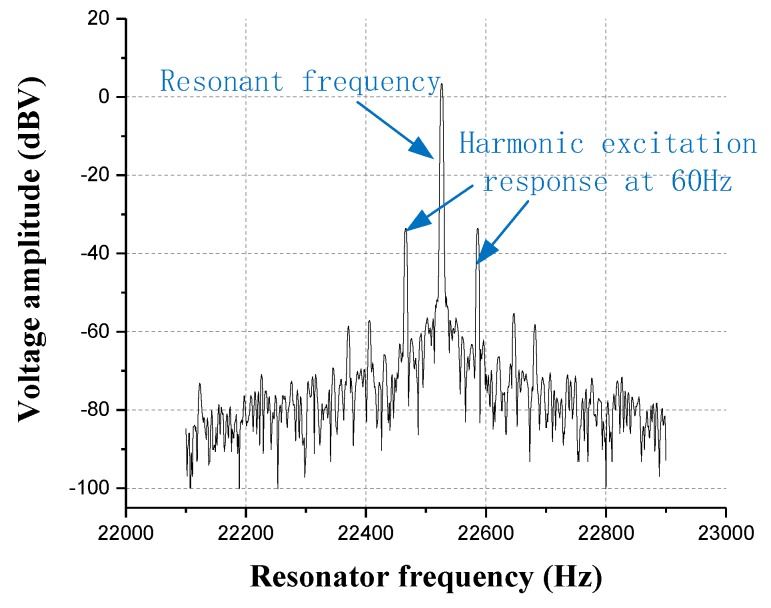
The amplitude-frequency characteristic curve of the resonator excited by the oscillatory air flow at 60 Hz.

In order to implement the experiment of the oscillatory air flow velocity for the artificial lateral line system, an oscillatory air flow velocity with a frequency of 60 Hz is generated by the AC excitation using a speaker. An experiment of FFT is performed on the output frequency signal of resonator by a spectrum analyzer, as shown in Figure 18. Due to the excitation of the oscillatory air flow, a modulated signal is generated at the 60 Hz from the drive frequency of the resonator.

**Figure 19:**
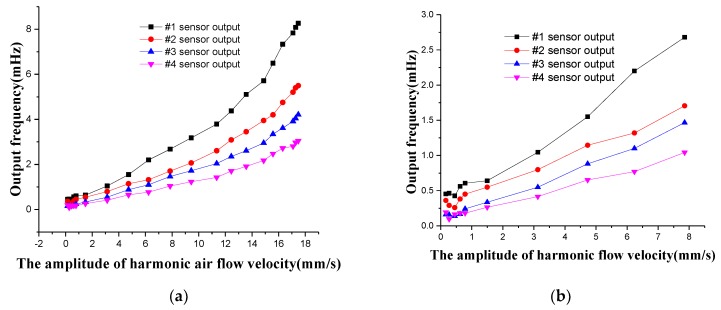

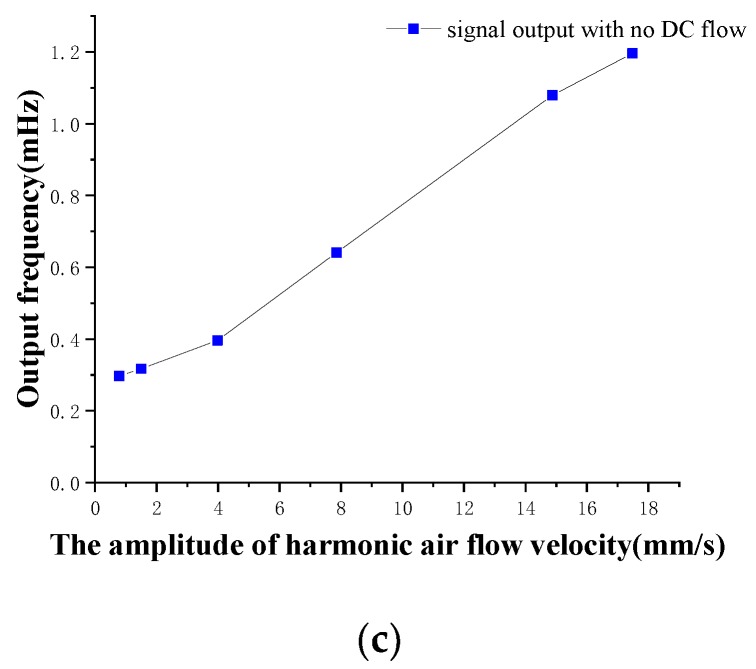
Output frequency response of the oscillatory air flow velocity for artificial lateral line system. (**a**) Output frequency signal of four superficial flow velocity sensors; (**b**) Partial amplification of Figure (**a**) in the oscillatory air flow velocity of 0–8 mm/s; (**c**) Output frequency signal of canal flow velocity sensor.

The oscillatory air flow is generated through the speaker, and the sound pressure is measured by the microphone and converted into the corresponding particle velocity of air flow. The amplitude of the oscillatory air flow is limited from 0 to 18 mm/s. In the FPGA, the LMSD algorithm is utilized to solve the oscillatory amplitude of the four superficial flow velocity sensors in the artificial lateral line system, shown in Figure 19. According to Figure 19a,b, the minimum threshold of the oscillatory flow velocity amplitude that can be measured by the artificial lateral line system is 0.785 mm/s. When the amplitude of air vibration flow is less than the corresponding amplitude of the threshold, the oscillatory signal will be covered up by the surrounding noise signal. As a result, the sensor can’t detect the effective oscillatory signal. The threshold is obtained by converting the amplitude of this threshold into the particle vibration velocity. The output of the canal artificial lateral line system is the superimposed data of four canal flow velocity sensors, shown in Figure 19c. The corresponding output of the canal artificial lateral line system is 1.2 mHz in the excitation of the oscillatory air flow velocity of 18 mm/s, which is due to the attenuation phenomenon caused by high-pass filtering of the canal artificial lateral line system.

### 4.3. Excitation of Superimposed Constant and Oscillatory Air Flow Velocity

**Figure 20:**
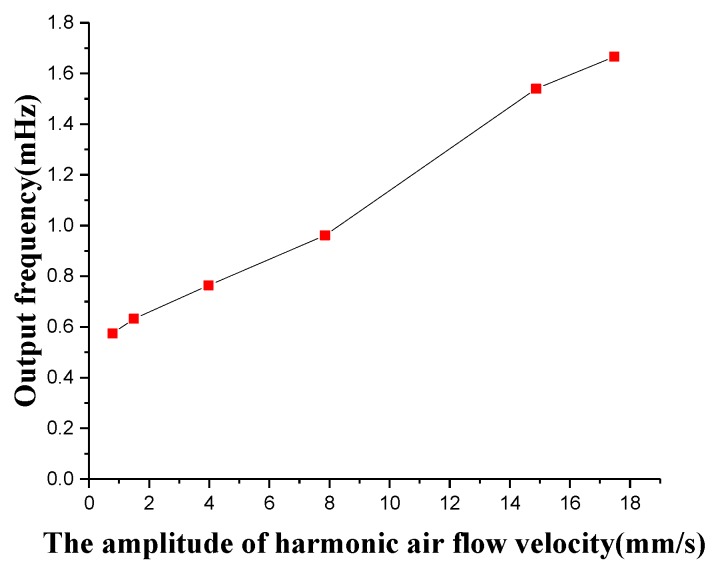
Output frequency signal of canal flow velocity sensor at the superimposed constant and oscillatory air flow velocity.

Simultaneously, the superficial and canal artificial lateral line systems are tested for the superimposed constant and oscillatory air flow velocity. The constant air flow velocity of 2 m/s and the oscillatory air flow velocity from 0 to 18 mm/s is superimposed to excite two artificial lateral line systems. The experimental results of the superficial artificial lateral line system indicate that the superficial artificial lateral line system can hardly measure the oscillatory air flow velocity due to the influence of the constant flow velocity, since it is almost fully submerged in the constant air flow velocity. The output frequency response of the oscillatory air flow velocity which is measured by the canal artificial lateral line system under the influence of strong constant air flow velocity is shown in Figure 20. The canal artificial lateral line system is capable of filtering the constant or low-frequency air flow velocity and measuring a weak oscillatory air flow velocity signal. Compared with Figure 19c, the amplitude of the oscillatory air flow velocity measured by the canal artificial lateral line system becomes larger because of the presence of constant air flow velocity, which increases the pressure difference between the canal holes. A probable reason is that the constant flow rate may have some resonance components and noise, which increase the amplitude of the demodulated oscillatory signal.

## 5. Conclusions

In this paper, an artificial lateral line system based on bionic hair sensor with resonant readout is studied. The artificial lateral line system can be employed to measure the air flow rate and weak oscillatory flow velocity. First, the sensitive mechanism of the two kinds of sensors in the artificial lateral line system is analyzed, and the structural design and finite element simulation are implemented. The finite element simulation results verify the correctness of the theory and structure design. Then the control circuit of the artificial lateral line system is designed, employing a demodulation algorithm of oscillatory signal based on the least mean square error algorithm which is used to calculate the oscillatory air flow velocity. Finally, the experiments are implemented to assess the performance of two artificial lateral line systems. The experimental results indicate that the two artificial lateral line systems can be applied for the measurement of the constant and oscillatory air flow velocity. The maximum scale factor of air flow velocity measurement is 6.97 Hz/(m/s)^2^, while the minimum threshold of oscillatory flow velocity measurement is 0.785 mm/s. Moreover, the artificial canal neuromast lateral line system can filter out low-frequency disturbance and has good sensitivity for high frequency flow velocity. The artificial lateral line system based on the bionic hair sensor with resonant readout has high sensitivity and a low threshold; not only can it accurately measure the constant air flow rate, but it can also measure the amplitude of the oscillatory air flow velocity through the internal demodulation algorithm, which lends it very broad application prospects.

## Figures and Tables

**Table 1:** Structure parameters.

Parameter (Unit)	Value
Hair height *L*_H_ (µm)	3000/6000/9000
Hair diameter *R* (µm)	1000
Hair density *ρ* (g/cm^3^)	1.05
Natural frequency of resonant beam *f*_0_ (kHz)	20
Drag coefficient *C_D_*	0.62
Coefficient related to the resonator *λ*	0.14
Magnification time *A*	7.06
Attenuation coefficient *η*	0.81
Resonator beam length *L* (µm)	1300
Resonant beam width *w* (µm)	10
Resonant beam thickness *h* (µm)	100

**Table 2:** Sensor parameters and system simulation parameters.

Parameter (Unit)	Value
Natural frequency of resonator *ω_d_* (rad/s)	22000 × 2π
Quality factor of resonator *Q*	42
Damping ratio of resonator *ξ*	0.0119
Proof mass of resonator *m* (kg)	8 × 10^−9^
Damping coefficient of resonator *c* (Ns/m)	2.63 × 10^−5^
Stiffness coefficient of resonator *k* (N/m)	152.86
The cutoff frequency of low pass filter *f_c_* (Hz)	75
System sampling time *T_s_* (s)	1 × 10^−6^
Proportion factor of PI(1) in amplitude closed loop *K_p_*_1_	0.1
Integral factor of PI(1) in amplitude closed loop *K_i_*_1_	2.2 × 10^−4^
Loop filter coefficient *C*_1_	0.01
Loop filter coefficient *C*_2_	0.001
VCO sensitivity (Hz/V)	50
Initial frequency of VCO (Hz)	21860
Torque coefficient of *F_d_* in the module ‘*F*_cn_’	5.664 × 10^−9^
